# Arhgap17, a RhoGTPase activating protein, regulates mucosal and epithelial barrier function in the mouse colon

**DOI:** 10.1038/srep26923

**Published:** 2016-05-27

**Authors:** So-young Lee, Hwain Kim, Kyoungmi Kim, Hyunji Lee, Seungbok Lee, Daekee Lee

**Affiliations:** 1 Department of Life Science, Ewha Womans University, Seodaemun-gu Ewhayeodae-gil 52, Seoul, 03760, S. Korea; 2 Department of Dental Science, Seoul National University, Jongro-gu Daehak-ro 101, Seoul, 08826, S. Korea

## Abstract

Coordinated regulation of the actin cytoskeleton by the Rho GTPase family is required for the maintenance of polarity in epithelial cells as well as for their proliferation and migration. A RhoGTPase-activating protein 17 (Arhgap17) is known to be involved in multiple cellular processes *in vitro*, including the maintenance of tight junctions and vesicle trafficking. However, the function of Arhgap17 has not been studied in the physiological context. Here, we generated Arhgap17-deficient mice and examined the effect in the epithelial and mucosal barriers of the intestine. Reporter staining revealed that Arhgap17 expression is limited to the luminal epithelium of intestine. Arhgap17-deficient mice show an increased paracellular permeability and aberrant localization of the apical junction complex in the luminal epithelium, but do not develop spontaneous colitis. The inner mucus layer is impervious to the enteric bacteria irrespective of Tff3 downregulation in the Arhgap17-deficient mice. Interestingly however, treatment with dextran sulfate sodium (DSS) causes an increased accumulation of DSS and TNF production in intraluminal cells and rapid destruction of the inner mucus layer, resulting in increased severity of colitis in mutant mice. Overall, these data reveal that Arhgap17 has a novel function in regulating transcellular transport and maintaining integrity of intestinal barriers.

Intestinal epithelial cells (IECs) serve a fundamental role in absorbing nutrients and preventing the systemic penetration of harmful substances in the gastrointestinal tract. To conserve epithelial integrity, two barriers have been established: a mucosal barrier consisting of a mucus layer outside the apical surface of IECs and an epithelial barrier connecting an apical junction complex (AJC) proteins between the adjacent epithelial cells^1^. The failure of either barrier causes various forms of intestinal pathophysiology, including inflammatory bowel diseases (IBDs)^2^,^3^,^4^. The mucus layer includes the inner and outer mucus layers containing heavily glycosylated mucins secreted from intestinal goblet cells^3^. The inner mucus layer works as a physical barrier that prevents the bacterial penetration in the lumen, whereas the outer mucus layer provides a habitat for commensal microorganisms^5^. Genetic loss of Muc2, the major component of the mucus, causes spontaneous colitis in mice, indicating the importance of the mucosal barrier to the enteric bacteria^6^. In the DSS-induced ulcerative colitis (UC), the penetration of bacteria into the inner mucus layer long before the beginning of inflammation occurs, suggesting that DSS may alter the property of mucus directly^7^. The character of the inner mucus layer is also closely associated with the pathological conditions of both Il10-null mice and patients with UC^8^.

The AJC, including tight junction (TJ) and adherens junction (AJ), in the IECs plays a major role in the formation and maintenance of epithelial barriers. TJs consist of the transmembrane adhesion proteins claudins, occludin, JAMs, tricellulin and the key cytoplasmic scaffolding proteins ZO–1, −2, and −3 9. Homophilic adhesion of transmembrane proteins between adjacent cells limits paracellular diffusion and regulates selective transport. ZO proteins organize TJ by connecting transmembrane proteins to the actin cytoskeleton. AJs consist of E-cadherin, a major transmembrane adhesion protein, and cytoplasmic adaptors including p120-catenin, *β*-catenin, and *α*-catenin which link the cytoplasmic tail of E-cadherin to the actin cytoskeleton^2^. AJs provide mechanically adhesive links between the adjacent cells and also help to assemble TJ. Disruption of the epithelial barrier due to AJC dysfunction resulting in the infiltration of bacterial products, dietary antigens and microorganisms across the epithelium leads to intestinal pathogenesis^4^.

The formation and maintenance of the AJC is regulated by numerous factors, including the Rho GTPase family^10^. Rho GTPase family members including the Cdc42, Rac, and Rho subfamilies, work as molecular switches in many cellular processes accompanying cytoskeletal organization^11^. The activity of Rho GTPases is tightly regulated by guanine nucleotide exchange factors (GEFs) and Rho GTPase activating proteins (GAPs), which respectively generate GTP-bound active and GDP-bound inactive GTPases^11^. Gene targeting has revealed the new function of Rho GTPases and regulators in a physiological context^12^,^13^. IEC-specific deletion of Cdc42 in mice affects the proliferation, differentiation, migration and polarity of cells, therefore these changes together result in severe impairments of intestinal epithelial barrier and homeostasis^14^. The significance of GAP regarding the intestinal barrier function is based on the studies on Myosin IXB (MYO9B), a single-headed molecular motor with GAP activity. Genetic variations of MYO9B are highly correlated with IBDs^15^,^16^, and the defect in Rho-mediated actin cytoskeletal organization in maintaining TJ may contribute to MYO9B-related IBDs^17^.

Arhgap17 (also known as Rich1) was initially identified as a neuron-specific GAP involved in regulated exocytosis^18^. Soon after, another group revealed that it is a Cip4-binding RhoGAP expressed broadly in human tissues^19^. In both studies, they showed that Arhgap17 modulates Cdc42 and Rac1 activity. Recently, it was revealed that Arhgap17 contributes to the maintenance of TJ by modulating Cdc42 activity via binding with the TJ scaffolding protein angiomotin^20^. Moreover, Arhgap17 mediates the function of merlin tumor suppressor by downregulating Rac1 activity after being released from the angiomotin-Arhgap17 complex in the TJ^21^. Compared to *in vitro* studies, the Arhgap17 function is far less defined in physiological contexts, except for the finding that dRich, a *Drosophila* orthologue of Arhgap17, modulates synaptic growth and function by regulating Gbb secretion in postsynaptic cells of the fly^22^. To further reveal the *in vivo* function of Arhgap17, we generated Arhgap17-deficient mice and investigated the hypothesis that Arhgap17 may regulate the epithelial barriers in the intestine. We found that the loss of *Arhgap17* expression in the intestinal tract results in the partial mislocalization of AJC proteins with a slight increase in paracellular permeability but does not lead to spontaneous colitis. However, the Arhgap17-deficient mice exhibited increased susceptibility to DSS-induced colitis likely associated with a rapid accumulation of DSS in the intraluminal cells and with increased vulnerability of the inner mucus layer. These results indicate that Arhgap17 plays an important role in regulating transcellular transport and mucosal barrier and that Arhgap17-deficient mice represent a useful model in studying UC.

## Results

### Arhgap17 mutant mice show normal development

**Figure 1:**
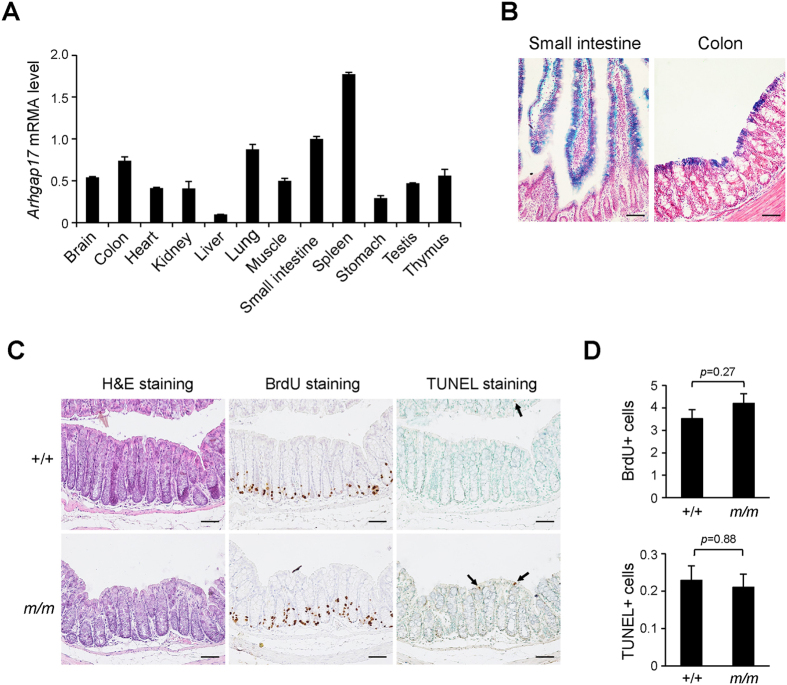
Expression of Arhgap17 in the luminal epithelium of the intestine. (**A**) qRT-PCR analysis of *Arhgap17* mRNA level in various tissues of 129/S6 mice. The relative amount of *Arhgap17* mRNA was compared to that of the small intestine. (**B**) Endogenous Arhgap17 expression was examined by LacZ staining in the intestine of Arhgap17-deficient mice. (**C**) Colon sections were stained with H&E, immunostained with an anti-BrdU antibody or analyzed with the TUNEL assay. (**D**) The number of BrdU-positive cells or TUNEL-positive cells (arrows) per crypt are shown (BrdU, n = 4; TUNEL, n = 6). +/+, wild-type; *m/m*, Arhgap17-deficient mice. Scale bar, 50 μm.

Data regarding generation of *Arhgap17* mutant mice and loss of Arhgap17 expression in mutant mice are described in the Supplementary Materials and Supplementary Fig. 1. *Arhgap17* transcripts were expressed ubiquitously (Fig. 1A). Tissue- and cell type-specific *Arhgap17* gene expression was analyzed by histological examination of LacZ activity. LacZ staining was detected widely in the developing embryo, but it was limited in the subregion of adult tissues (Supplementary Fig. 2A,B). Intense staining was observed in the stratified squamous epithelium of the esophagus and stomach. In the kidney and testis, relatively moderate staining was detected in a subpopulation of cells, whereas punctate LacZ staining was observed in the hippocampus, heart, lung, muscle and skin. Interestingly, moderate LacZ staining was only detected in the luminal epithelium of the intestine but not in the cryptic epithelium (Fig. 1B). The expression pattern in the gut suggests that Arhgap17 is likely to be involved in the physiology of the intestinal epithelium. However, the overall histology was similar in the colon between wild-type and Arhgap17-deficient mice (Fig. 1C), except that some enlarged goblet cells were found in the ileum. The number of BrdU- and TUNEL-positive cells in the colon (Fig. 1C,D) and ileum (Supplementary Fig. 3A,B) of Arhgap17-deficient mice was comparable to those in the wild-type mice, respectively. Only a slight increase in migration of IECs was observed in Arhgap17-deficient mice (Supplementary Fig. 3C,D). Despite the broad expression of the gene, Arhgap17-deficient mice did not show an overtly abnormal phenotype under normal physiological conditions, except that male had slightly less body weight gain (Supplementary Fig. 4).

### Arhgap17-deficient mice are more susceptible to DSS-induced colitis

**Figure 2:**
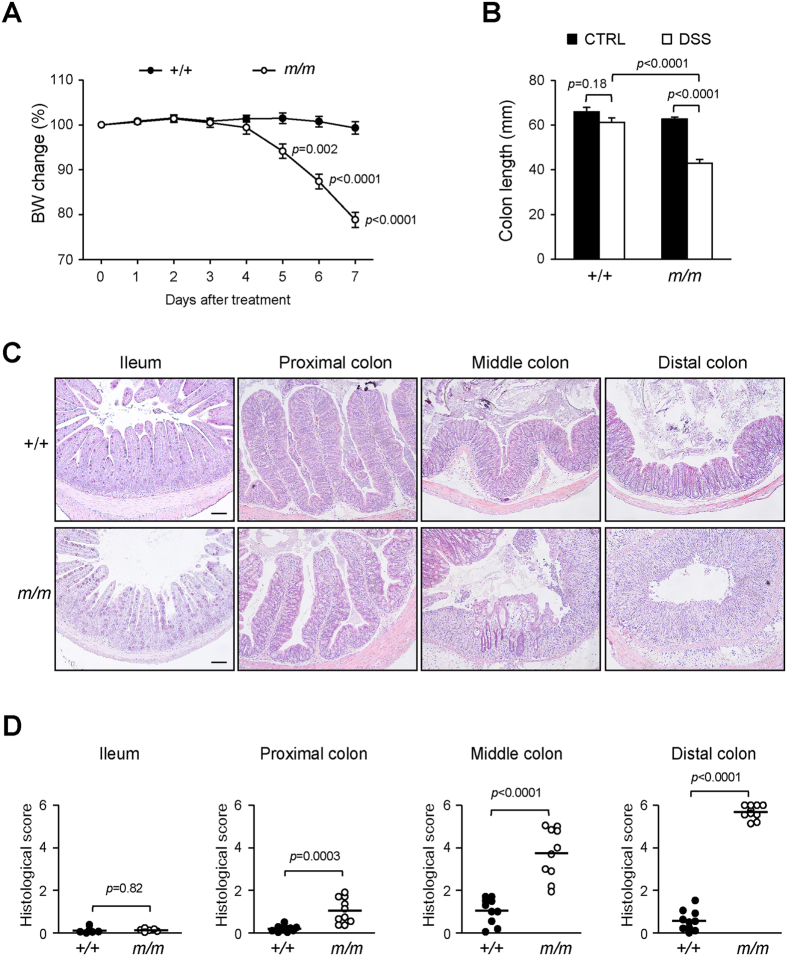
Effect of Arhgap17-deficiency on response to DSS treatment. (**A**) The body weight change expressed daily as a percentage of the body weight on the first day of exposure to DSS. (**B**) The length of the colon and, (**C**) cross section of the ileum and colon 7 days after treatment was examined with H&E staining. Scale bar, 100 μm, 100X magnification. (**D**) Each dot represents the histological damage score of an individual mouse. Ileum, n = 6; colon, n = 10.

Arhgap17 is unlikely to be essential for the intestinal homeostasis under normal physiological conditions. We therefore analyzed Arhgap17-deficient mice after DSS treatment. Colitis severity was analyzed after continuous exposure to 1.5% DSS in drinking water, a concentration lower than the typical concentration that induces colitis in wild-type mice. A significant body weight loss started to occur after five days of exposure to DSS in Arhgap17-deficient mice, whereas wild-type mice showed no body weight loss even after seven days (Fig. 2A). The consumption of DSS-supplemented water was not different between two groups for the first three days, but Arhgap17-deficient mice consumed less DSS-water subsequently. The colon length was similar in both genotypes before treatment, but the length was shortened significantly in mutant mice by DSS treatment (Fig. 2B). Severe lesions were found toward the distal region of the colon in mutants (Fig. 2C) resulting in higher histological damage scores in mutant mice (Fig. 2D). However, lesions in the ileum were rare in both genotypes (Fig. 2C,D). Additionally, LacZ staining in the colonic epithelial cells was attenuated due to the disintegration of epithelial cells after the DSS treatment (Supplementary Fig. 5A).

**Figure 3:**
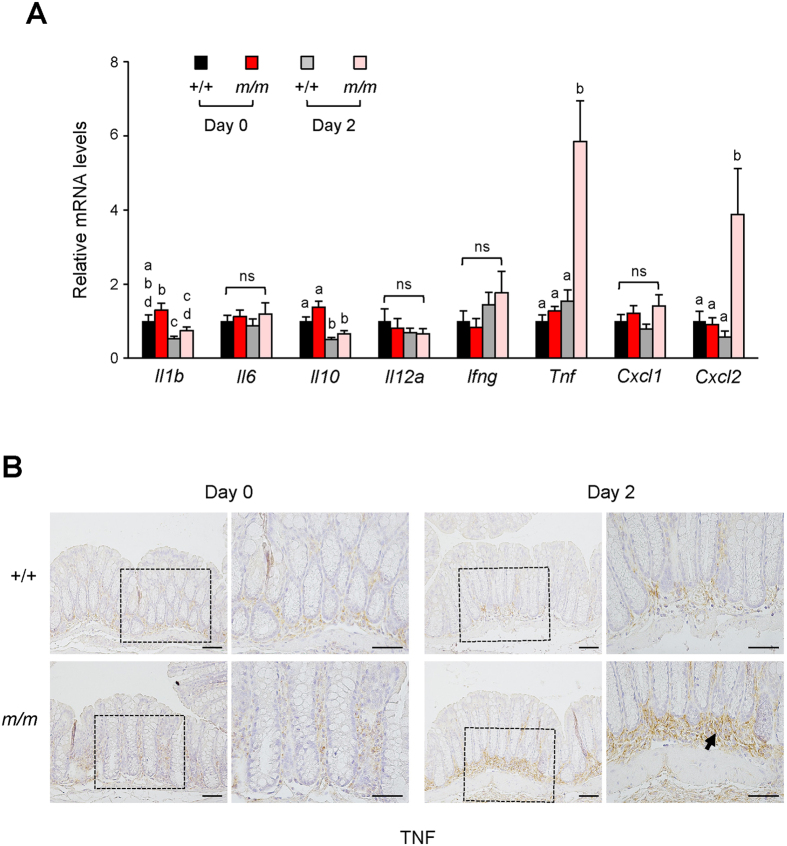
Analysis of inflammatory cytokines and chemokines gene expression after DSS treatment. (**A**) The relative amount of each mRNA was determined by qRT-PCR analysis and compared to that of the wild-type mice before treatment (Day 0) or after DSS treatment for 2 days (Day 2). n = 8–11. Bars with different letters are significantly different (two-tailed unpaired *t*-test, *p* < 0.05), ns indicates a non-significant result. (**B**) The distal colon sections were immunostained with an anti-TNF antibody. Left, 200X magnification; Right, 400X magnification of a rectangle. Arrow indicates strong staining in the submucosal area. Scale bar, 50 μm.

To analyze the mechanism of the earlier onset of DSS-induced colitis in mutants, the gene expression of inflammatory cytokines and chemokines was examined in the colon two days after the treatment. There was no difference in the mRNA levels between the wild-type and Arhgap17-deficient mice before treatment (Fig. 3A). However, the mRNA levels of *Tnf* and *Cxcl2*, both produced by macrophages^23^,^24^, were significantly increased only in DSS-treated Arhgap17-deficient mice, indicating that an inflammatory response was already initiated. Whereas the mRNA levels of *Il1b* and *Il10* were decreased in both genotypes after treatment, the other mRNA levels remained constant regardless of treatment or genotype. Consistent with the sharp increase in *Tnf* mRNA, the immunostaining of the anti-TNF antibody revealed that a subset of the intraluminal cells contained high levels of TNF protein in Arhgap17-deficient mice (Fig. 3B). However, cell proliferation, apoptosis, and cell migration were similar between colonic cells of wild-type and Arhgap17-deficient mice two days after DSS treatment (Supplementary Fig. 5B,C).

### Arhgap17 maintains the proper barrier junction proteins

**Figure 4:**
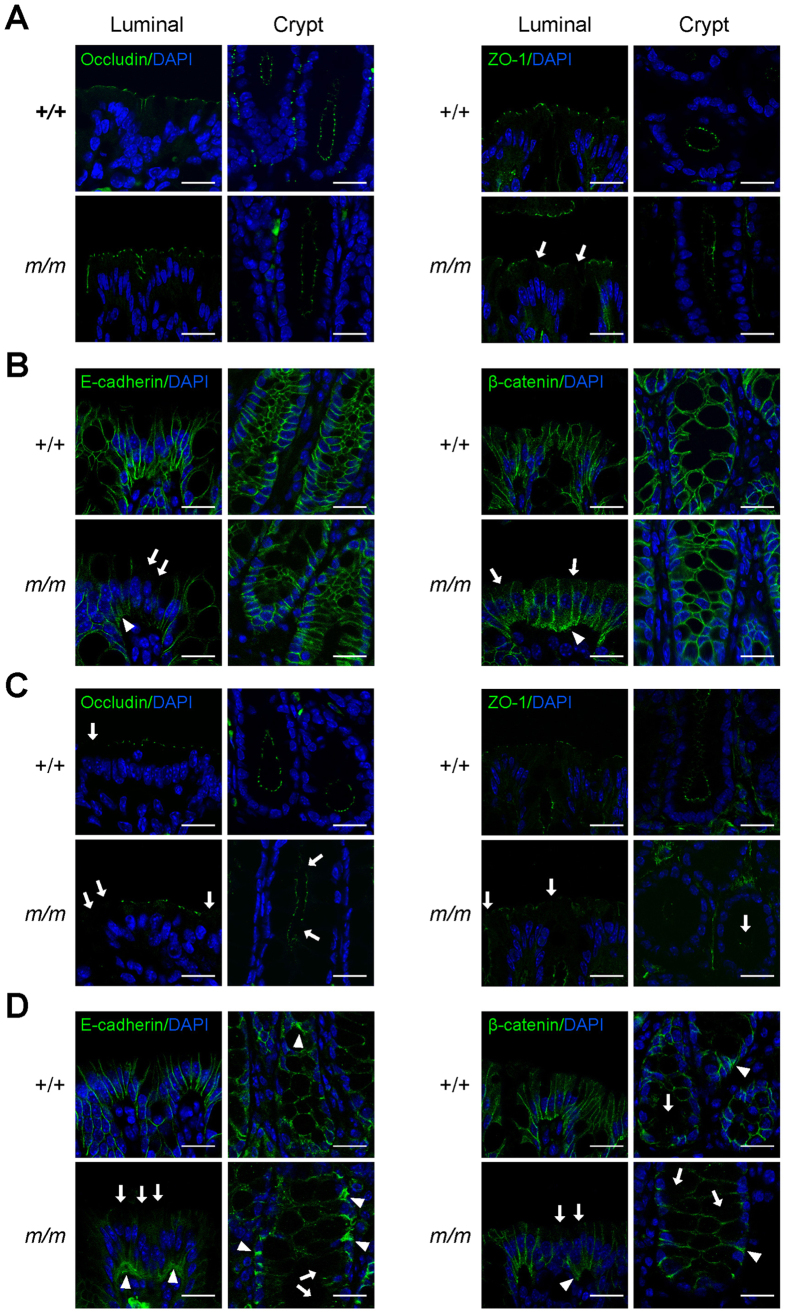
Immunofluorescence analysis of AJC proteins localization in the colon. Immunofluorescence staining of AJC proteins in the distal colon (**A**,**B**) before or (**C**,**D**) after DSS treatment for 2 days. (**A**,**C**) Sections were immunostained with the indicated antibody. Arrows indicate the mislocalization of TJ proteins in the apical/lateral boarder of epithelial cells, n = 3–4. (**B**,**D**) Sections were immunostained with the indicated antibody, n = 5–6. Arrows indicate the loss of AJ proteins in the apical/lateral boarder of epithelial cells, whereas arrowheads indicate lumps of proteins in the basolateral epithelium. The luminal epithelium is shown on the left (Luminal); the cryptic epithelium is shown on the right (Cryptic). Scale bar, 20 μm.

Arhgap17 maintains the integrity of TJ in epithelial cells by modulating the Cdc42 *in vitro*^20^, suggesting that it may also regulate the polarity of IECs *in vivo*. We determined the localization of TJ and AJ proteins in the gut by immunofluorescence staining. The distribution of occludin was comparable between the wild-type and Arhgap17-deficient mice in the colonic epithelium (Fig. 4A) or ileum (Supplementary Fig. 6A), irrespective of the genotype. Interestingly, the distribution of ZO-1 was marginally disrupted only in the colonic luminal epithelium of Arhgap17-deficient mice but not in the colonic cryptic epithelium. Similarly, partial defects were seen in the ZO-1 localization only in the villus but not in the crypt of mutant ileum (Supplementary Fig. 6A). In Arhgap17-deficient mice, some luminal epithelial cells showed partially down-regulated E-cadherin and *β*-catenin staining at the apical/lateral boarder (Fig. 4B). Moreover, clumps containing high levels of E-cadherin and *β*-catenin were frequently detected in the basal region of the Arhgap17-deficient mice. However, these aberrant localizations of AJ proteins were not observed in the cryptic epithelium. Similarly, the localization of AJ proteins was recapitulated in the ileum (Supplementary Fig. 6B). The disappearance of E-cadherin staining in the apical/lateral boarder was more clearly seen by co-immunostaining with F-actin, an apical membrane marker, in the IECs of Arhgap17-deficient mice (Supplementary Fig. 7A,B).

The TNF-mediated increase in intestinal permeability is an important pathogenic factor in the development of intestinal inflammation^4^,^25^. Because marked induction of TNF producing cells after DSS treatment in Arhgap17-deficient mice may cause a disruption of the intestinal epithelial TJ barrier, we analyzed the localization of TJ and AJ proteins two days after the treatment. A partial loss of occludin and ZO-1 (Fig. 4C) and more severe mislocalization of E-cadherin and *β*-catenin in the epithelium of Arhgap17-deficient mice were clearly seen as the result of DSS treatment (Fig. 4D). In contrast, the integration of TJ and AJ proteins remained largely intact in the wild-type mice only with a slightly aberrant distribution. Likewise in the ileum, DSS treatment resulted in aberrant TJ and AJ protein localization in Arhgap17-deficient mice but not in wild-type mice (Supplementary Fig. 6C,D).

### Arhgap17-deficient mice have increased intestinal permeability

**Figure 5:**
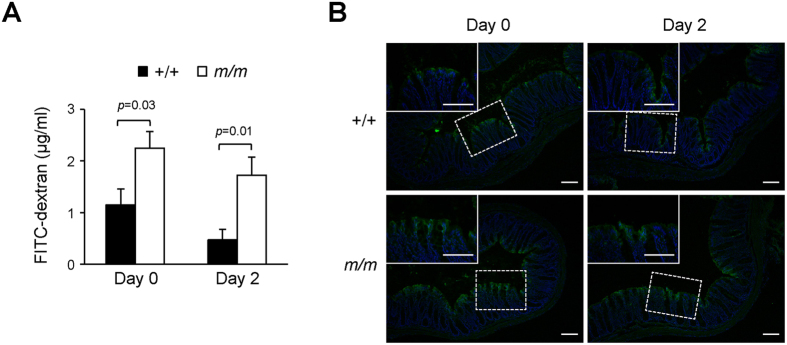
Effect of Arhgap17-deficiency on intestinal permeability. *In vivo* intestinal permeability was examined before and after DSS treatment for 2 days, respectively. (**A**) The serum concentration of FITC-dextran was measured, n = 7–9. (**B**) Fluorescence microscopic images were examined for the location of FITC-dextran in the distal colon after gavage, n = 9. Original magnification, 100X; Top left, 400X magnification of dashed rectangle. Scale bar, 50 μm.

Given that mislocalization of TJ proteins leads to increased intestinal permeability, we sought to test if Arhgap17-deficiency has similar effect. This was done by measuring the serum FITC-dextran concentration after gavage. The levels of FITC-dextran in the serum of Arhgap17-deficient mice were 2-fold higher than that in the wild-type mice (Fig. 5A), indicating an increase in intestinal permeability. Despite a slight increase in TJ protein mislocalization two days after DSS treatment, the serum concentration of FITC-dextran was comparable to that of non-treated Arhgap17-deficient mice. Consistently, fluorescent microscopic analysis showed deeper infiltration of FITC-dextran into the colonic epithelium of Arhgap17-deficient mice compared to the wild-type mice but a similar extent with or without DSS treatment (Fig. 5B). That no significant increase in epithelial permeability was seen after DSS treatment in Arhgap17-deficient mice despite a dramatic TNF induction indicates that TNF-mediated disruption of intestinal TJ barriers is not an initial event of the DSS-induced colitis in these mice.

### Increased intraluminal DSS accumulation in the Arhgap17-deficient mice

**Figure 6:**
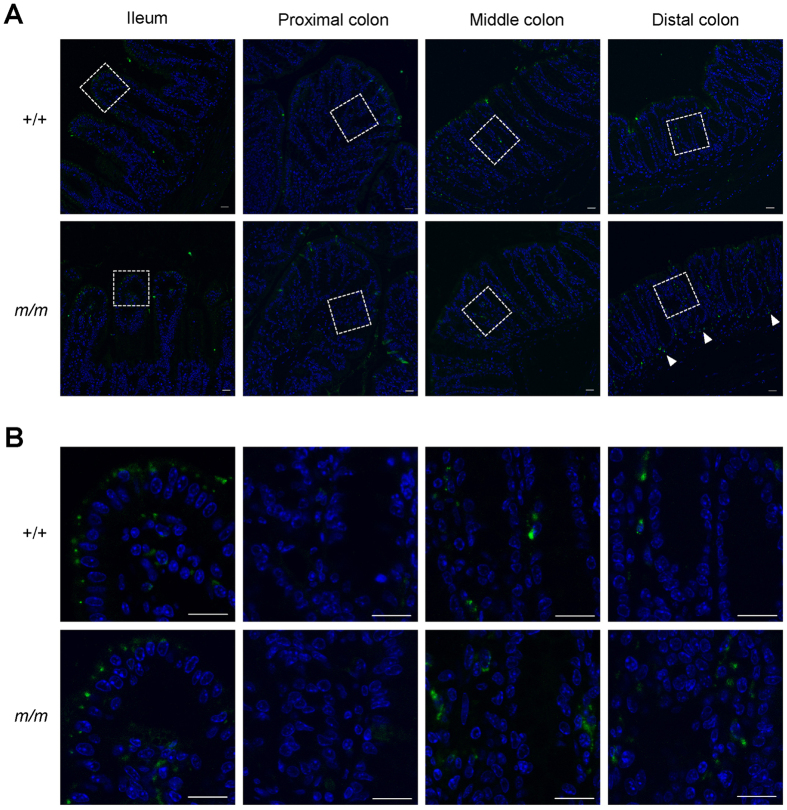
Intestinal DSS distribution after FITC-DSS treatment. DSS distribution was examined after FITC-DSS treatment for 24 h using confocal microscope, n = 4. (**A**) Representative fluorescence microscopic images are shown, 400X magnification. Arrowheads indicate DSS deposits in the lamina propria cells at the bottom part of the mucosa. (**B**) 1000X magnification of dashed rectangle. Scale bar, 20 μm.

It appears that the significant induction of TNF in the Arhgap17-deficient mice by DSS treatment has no effect on the intestinal permeability at least initially. DSS treatment in the guinea pig showed a selective uptake of DSS by the epithelial cells and subsequent accumulation in the intraluminal macrophages^26^, indicating that DSS passes through epithelial cells and diffuse into lamina propria. Therefore we examined the localization of DSS in the mucosa one day after DSS treatment using FITC-DSS (Fig. 6A,B). In the proximal colon, DSS-positive luminal IECs were more frequently found in Arhgap17-deficient mice, but DSS-positive cells were rare in the intraluminal region of both genotypes. In the middle and distal colon, DSS was detected both in the luminal IECs and intraluminal cells irrespective of the genotype. However, DSS-positive cells were markedly increased at the bottom of mucosa in the distal colon of Arhgap17-deficient mice. In the ileum, both genotypes exhibited punctate DSS accumulation in the IECs of the villus but not of the crypt. These results suggest that increased accumulation of DSS in the intraluminal cells is likely associated with a dramatic TNF induction and the subsequent colitis in the Arhgap17-deficient mice.

### Arhgap17-deficient mice have intact mucosal barrier under normal physiological conditions

**Figure 7:**
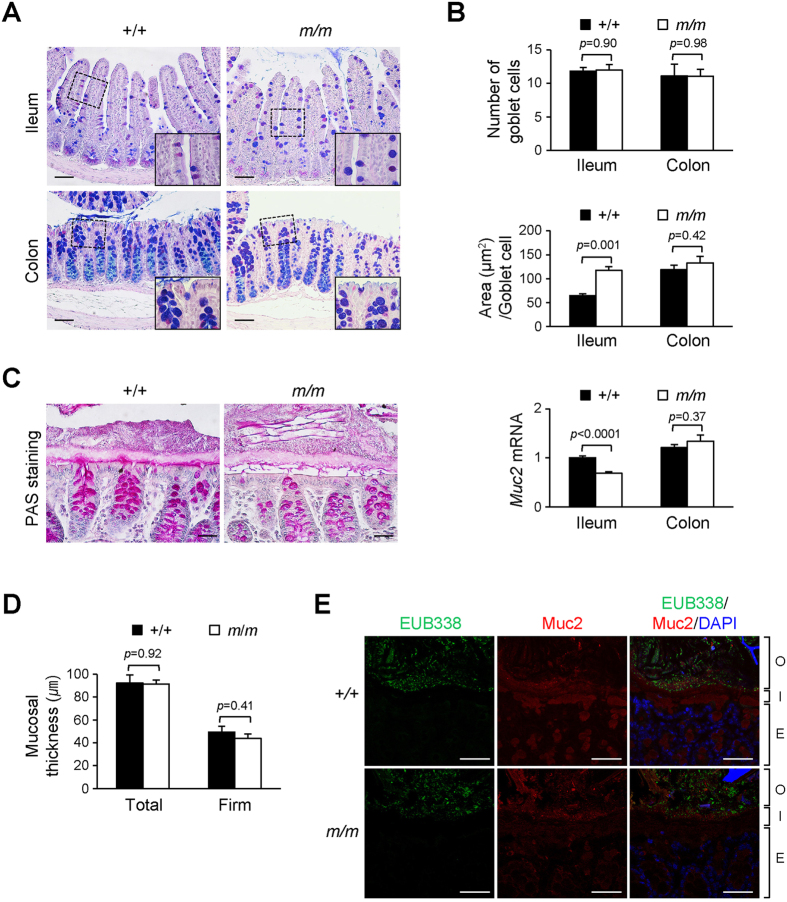
Analysis of mucus layer under normal physiological conditions. (**A**) AB/PAS staining of mucins in the ileum (Top) and distal colon (Bottom). Original magnification, 200X; Bottom right, 1000X magnification of dashed rectangle. (**B**) Top, the number of goblet cells per villus (ileum) or per crypt (colon); Middle, the area of mucin vesicles; Bottom, the *Muc2* mRNA level. (**C**) PAS staining to examine the mucus layer is shown. (**D**) The total and firm *in vivo* mucus thickness in the distal colon. (**E**) The mucus layer in the distal colon was analyzed by hybridization with EUB338 probe, followed by immunostaining with anti-Muc2 antibody and DAPI staining. (**B**) Top, n = 8–11; Middle, n = 8–9; Bottom, 7–9; (**D**) n = 9–10; (**E**) n = 4. O, outer mucus layer; I, inner mucus layer; E, epithelial layer. Scale bar, 50 μm.

To determine the relevance of Arhgap17 in mucin secretion, we histologically examined goblet cells and the integrity of the mucus layer. The number of goblet cells determined by AB/PAS staining was similar in the ileum or colon of Arhgap17-deficient mice compared to that in the wild-type mice (Fig. 7A,B). Interestingly, Arhgap17-deficient mice showed more enlarged mucin-stained vesicles in the ileum, but not in the colon. The mRNA expression levels of Muc2 showed a slight reduction only in the ileum, but not in the colon of Arhgap17-deficient mice. PAS staining showed that the inner mucus layers in the colon were of comparable thickness between the two groups (Fig. 7C). Neither the total nor the firm mucus was affected in terms of thickness by Arhgap17 deficiency (Fig. 7D). The integrity of the mucus layer was further examined with *in situ* hybridization using a general 16S rRNA probe for the presence of bacteria and immunostaining with anti-Muc2 antibody in the colon (Fig. 7E). There were no bacteria in the inner mucus layer of either wild-type or Arhgap17-deficient mice, indicating that the mucosal barrier function was independent of Arhgap17 in normal physiological conditions.

### DSS induces rapid destruction of mucosal barrier in the Arhgap17-deficient mice

**Figure 8:**
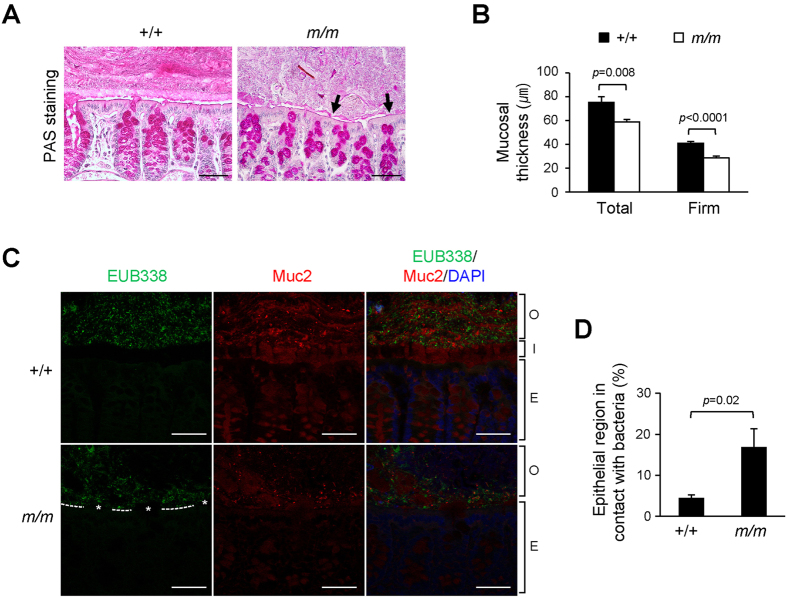
Effect of DSS treatment for two days on the colonic mucus layer. (**A**) PAS staining is shown. Arrows indicate eroded areas of the mucus layer. (**B**) Total and firm *in vivo* mucus thickness was determined. (**C**) The mucosal barrier was analyzed by hybridization with the EUB338 probe, followed by immunostaining with the anti-Muc2 antibody and DAPI staining. Asterisks indicate the residual inner mucus layer attached to the epithelial cells. Dashed lines indicate areas in contact with bacteria. O, outer mucus layer; I, inner mucus layer; E, epithelial layer. (**D**) The proportion of epithelial areas in contact with bacteria was evaluated. (**B**) n = 7–8; (**C**) n = 7; (**D**) n = 7. Scale bar, 50 μm.

**Figure 9:**
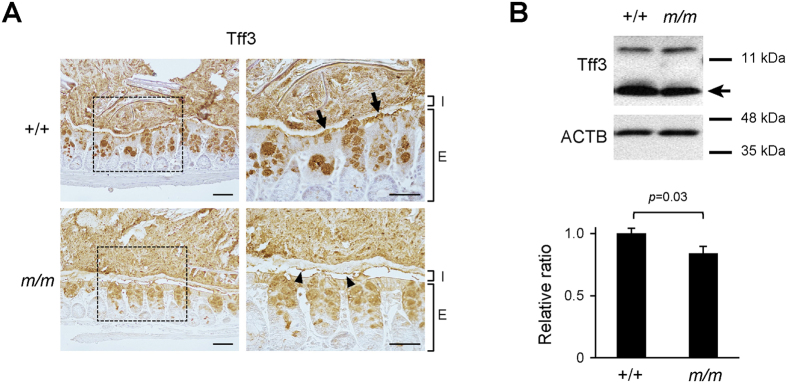
Analysis of Tff3 expression under normal physiological conditions. (**A**) The distal colon sections were immunostained with an anti-Tff3 antibody. Original magnification, 200X; right, 400X magnification of the dashed rectangle. I, inner mucus layer; E, epithelial layer. Arrows indicate areas stained thickly in wild-type, whereas arrowheads indicate areas stained thinly in Arhgap17-deficient mice. (**B**) Western blotting of the Tff3 protein in the distal colon. The relative band intensities of Tff3 were normalized to ACTB levels, n = 10. Arrow indicates specific Tff3 band.

An alteration of the mucus layer by DSS treatment results in bacterial infiltration into the inner mucus layer within 12 hours well ahead of inflammatory cell infiltration^7^. Therefore, we tested whether the mucus layer of Arhgap17-deficient mice is more susceptible to DSS treatment. After two days of the treatment, a partial erosion of the inner mucus layer occurred in all mice, but the erosion was more advanced in Arhgap17-deficient mice than in wild-type mice (Fig. 8A). Consistently, Arhgap17-deficient mice had thinner mucus layers than those of the wild-type mice (Fig. 8B). This applied to both the total mucus layer and the firm mucus layer. Moreover, EUB338 staining demonstrated a direct contact of IECs with bacteria in the eroded mucus (Fig. 8C, Supplementary Fig. 8A). Consistently, the percentage of epithelial region in contact with bacteria was significantly higher in Arhgap17-deficient mice than in wild-type mice (Fig. 8D). It has been reported that genetic loss of Tff3, a member of the trefoil family, results in the impairment of mucosal repair from DSS-induced colitis^27^. We thus performed immunostaining for Tff3 in the Arhgap17-deficient mice. In the colon of wild-type mice, relatively thick and rough Tff3 staining was observed in the bottom of the mucosal layer, which seemed to be attached to the luminal epithelium (Fig. 9A). In contrast, only a very thin layer of Tff3 staining remained in Arhgap17-deficient mice. This reduction in staining was accompanied by a detectable decline of protein levels (Fig. 9B) and a marked reduction of mRNA levels in Arhgap17-deficient mice (Supplementary Fig. 8B). These results suggest that Arhgap17 contributes to the maintenance of the intestinal mucus integrity through promoting proper expression and secretion of Tff3.

## Discussion

The dynamic organization of the actin cytoskeleton should be tightly regulated for cell division, migration, and vesicle trafficking of polarized IECs. The critical regulators of such processes, RhoGEFs and RhoGAPs that modulate the Rho GTPase family are not well known in IECs. Arhgap17, a RhoGAP was functionally analyzed *in vitro* using MDCK cells, a general model of epithelial cells that expressed a sufficient amount of Arhgap17 for western blotting^20^,^21^. Based on the interaction of Arhgap17 with angiomotin TJ scaffold protein and CIN85/CD2AP endocytic adaptors, the cellular function of Arhgap17 has been suggested to involve the intracellular trafficking of TJ proteins and the maintenance of TJ through the regulation of Cdc42 activity^20^. Here, we report a new set of related functions of Arhgap17 in the IECs that Arhgap17 contributes to the maintenance of AJC as well as the modulation of transcellular transport and the integrity of mucus layer.

A feature of IBDs is the increased paracellular permeability of the IECs^1^,^4^. As TJs play an essential role in the epithelial barrier, IEC-specific deletion of Claudin-7, a predominant intestinal claudin, enhances paracellular permeability and causes spontaneous colitis in mice^28^. To the contrary, occludin deficiency does not affect intestinal permeability in mice^29^, and the disruption of JAM-A does not induce spontaneous colitis despite the increase in intestinal permeability^30^,^31^. In contrast to the severe disruption of TJ only by the knockdown of Arhgap17 in MDCK cells^20^, both TJ and AJ showed partial defects in the luminal epithelium by the loss of Arhgap17, accompanying a slight increase in the paracellular permeability. Arhgap17-deficiency, however, was not enough to develop spontaneous colitis and disturb intestinal homeostasis, indicating the maintenance of epithelial barrier function in the absence of Arhgap17. It is intriguing to find a slight increase in IECs migration without the change in proliferation and apoptosis in Arhgap17-deficient mice. Aberrant localization of E-cadherin seems to be relevant to the increased migration of IECs in Arhgap17-deficient mice because IEC-specific *Cdh1* deletion causes enhanced migration^32^. Considering the absence of Arhgap17 expression in the cryptic epithelium, Arhgap17 is not likely involved in the initial formation of AJC. However, mislocalization of AJC proteins in the luminal epithelium of Arhgap17-deficient mice indicates that Arhgap17 is necessary for the proper maintenance of AJC.

The pathological features including severity of DSS-induced colitis depends on the molecular weight of DSS and the intestinal location^33^. Administration of 40 kDa DSS in mice induces colitis mainly in the distal and middle colon but rarely in the proximal colon. The initial mechanism of DSS-induced colitis is still uncertain, but a recent publication suggests that the formation of nano-lipocomplexes of DSS with medium-chain-length fatty acids plays a role in the delivery of DSS and subsequent damage to the colonic epithelial cells^34^. Beyond the DSS in IECs, DSS accumulation in the intraluminal cells of wild-type mice within one day after DSS treatment indicates the presence of transcellular DSS transport. Compared to the paracellular transport of small molecules, transcellular transport is known to play an important role in IECs for the uptake and secretion of macromolecules such as luminal antigens and secreted antibodies^35^. The finding of perinuclear DSS localization in the IECs is unexpected, indicating the uptake of DSS even in the ileum. It is quite interesting that deep penetration of DSS into the intraluminal cells occurs especially in the distal colon before a significant increase in epithelial permeability, and is correlated with the intestinal location of DSS-induced colitis and increased severity of colitis in Arhgap17-deficent mice. As suggested by the *in vitro* study, Arhgap17 may regulate transcellular transport of macromolecules in the IECs, and regional difference of transcellular transport of DSS is likely one of the factors for region-specific effect of DSS on colitis.

A recent publication has proposed that DSS-induced colitis begins with DSS-mediated change of mucus structures which allows bacteria to penetrate and disintegrate the inner mucus layer^7^. Muc2 mucin, together with other proteins, including Fcgbp and Tff3 secreted from goblet cells, constitutes the densely packed inner structural barrier that is impervious to commensal microorganisms^36^,^37^,^38^. Hence, the disintegration of the mucus layer is directly connected to intestinal pathogenesis^3^,^5^. Intestinal goblet cells maintain the mucus layer by secreting proteins through compound exocytosis^5^, which is coordinated by numerous proteins, including exocyst, SNARE, and the actin cytoskeleton complex^39^. However, it is not known how Rho GTPases regulate mucin secretion in goblet cells. That no change in the mucosal layer thickness is seen in Arhgap17-deficient mice indicates that Arhgap17 may not be involved in mucus secretion. Moreover, the inner mucus layer was functional at least as a barrier to the enteric bacteria in the Arhgap17-deficient mice. Rather, the increase in the vesicle size in the ileum of Arhgap17-deficient mice indicates that Arhgap17 may be involved with vesicle-vesicle fusion in the ileum.

Interestingly, Arhgap17-deficient mice showed increased sensitivity to DSS-induced colitis similar to *Tff3* knockout mice. Mice lacking Tff3 show reduced epithelial migration and increased DSS-induced mucosal injury without a change in mucin staining^27^. Luminal application of recombinant Tff3 attenuates injury, indicating extracellular action of Tff3^27^,^40^. Tff3 also forms a disulfide-linked heteromer with Fcgbp and contributes to the organization of the mucin network through an Fcgbp-Muc2 connection^36^,^37^. The finding of Tff3 localization mainly in the bottom of the inner mucus layer after secretion is unexpected. Although the Tff3 layer of the Arhgap17-deficient mice was much thinner than that in the wild-type mice, the inner mucus layer still can apparently act as a functional barrier to enteric bacteria. In contrast, the inner mucus layer of Arhgap17-deficient mice was more vulnerable to DSS, indicating an alteration of mucous properties. Considering the deleterious effects of DSS on IECs directly, there is a possibility that an increased severity of DSS-induced colitis in Arhgap17-deficient mice is due to the differential accumulation of DSS in IECs. However, the similar FITC-DSS accumulation and apoptotic index in IECs irrespective of the genotype indicates that the damage of IECs is not likely the initial event in DSS-induced colitis in Arhgap17-deficient mice. It is intriguing that the level of Tff3 in goblet cells was also decreased in Arhgap17-deficient mice. Considering the previous report showing that the severity of *Tlr2* knockout mice to DSS-induced colitis is associated with the reduction of Tff3 expression in goblet cells^40^, the increased sensitivity to DSS-induced colitis in Arhgap17-deficient mice could also be due to the reduction of Tff3 expression. We thus demonstrate a functional property of Arhgap17 whose loss of function in maintenance of the inner mucosal layer is likely a risk factor for UC.

## Methods

### The mice and histology

*Arhgap17* mutant mice were generated and maintained congenic on a C57BL/6J background (Supplementary Materials). All mice experiments were performed in accordance with the guidelines and were approved by the Institutional Animal Care and Use Committee at Ewha Womans University (Permit number: 2011-01-009). Age matched adult mice were used. The small intestine was divided into 10 equal parts and the most distal part next to the cecum was used as ileal tissue sample for analysis. The colon was divided into 3 equal parts, the proximal, middle, and distal region and the distal part was used for analysis except for the histological scoring after DSS treatment. For histology, intestines were fixed in 10% buffered formalin and embedded with paraffin. Rehydrated paraffin sections were stained with alcian blue (AB) solution and periodic acid Schiff (PAS) reagents according to the manufacturer’s protocol (Sigma-Aldrich) to stain goblet cells. The slides were further stained with hematoxylin. The number of goblet cells in the ileum was counted from more than 10 villi only with section in the middle of a villus. The area of each goblet cell was determined by measuring the size of vesicle with NIS-Elements BR 3.2 imaging software (Nikon) and Image J (imagej.nih.gov) program. The number and area of goblet cells in the distal colon was determined similarly except for analyzing goblets cells located within 0.1 mm from the luminal epithelium^8^,^36^.

### Reverse-transcription and quantitative PCR (qRT-PCR)

Total RNA preparation, reverse transcription, real-time PCR and quantification of mRNA were performed as described previously^41^, except that a KAPA SYBR FAST qPCR kit (KAPA Biosystems) and *GAPDH* or *18S rRNA* as an endogenous control were used. The primer sequences used for qRT-PCR are in the Supplementary Materials.

### DSS treatment and histological scoring

To induce colitis, 1.5% (w/v) DSS (36–50 kDa, MP Biomedicals) was treated as described previously^42^. The colon was collected seventh days after treatment and histological sections from the proximal, middle, and distal colon were stained with hematoxylin and eosinY (H&E). The severity of injury was graded as described in Supplementary Materials.

### Immunofluorescence and Immunohistochemistry

The ileum or distal colon was flushed with PBS and fixed in 10% buffered formalin. Immunofluorescence staining was performed as described previously^41^. Briefly, rehydrated paraffin or frozen sections were blocked using 5% normal goat serum or M.O.M mouse Ig blocking reagent (Vector Lab) and treated with primary antibody at 4 °C overnight. The sections were further incubated with secondary antibodies conjugated with Alexa 488 (Life Technologies), counterstained with DAPI and visualized with a LSM 510 Meta confocal microscope (Carl Zeiss). For immunohistochemistry, re-hydrated paraffin sections were treated with 3% H_2_O_2_ in deionized water and immunostaining was performed using Vectastatin ABC kit (Vector Lab) according to the manufacturer’s protocols. Antigen-antibody complexes were detected with a DAB peroxidase kit (Dako). For Tff3 immunohistochemistry, a sample of the distal colon with a fecal pellet was collected and fixed in methanol-Carnoy’s solution overnight^38^. The sources of the primary antibodies are in the Supplementary Materials.

### *In vivo* intestinal permeability

The epithelial barrier function of the intestine was assessed by measuring the serum concentration of FITC-dextran (4 kDa, Sigma-Aldrich) after gavage^43^. The mice fasted for 4 hours and were given 30 mg/ml of FITC-dextran (0.6 mg/g body weight). Blood was collected 4 hours later by cardiac puncture after CO_2_ euthanasia. The supernatant was collected by centrifugation at 3000 rpm for 10 minutes. The fluorescence in serum was measured by using a Multimode Detector DTX880 (Beckman Coulter), and the concentration was determined by comparison with a standard curve of serial-diluted FITC-dextran. For histology, the distal colon was thoroughly flushed with PBS and fixed with 4% paraformaldehyde in PBS at 4 °C overnight, and frozen sections were counterstained with DAPI. Images were examined using fluorescence microscope (ECLPISE 80i EPI, Nikon).

### Histological analysis of DSS distribution in the intestine

To examine the distribution of DSS in the mucosa, 1.5% (w/v) FITC-DSS (40 kDa, TdB Consultancy AB) was diluted to one-tenth with 1.5% (w/v) DSS (36–50 kDa, MP Biomedicals) and orally administered to mice *ad libitum* for 24 h. The ileum and colon were thoroughly flushed with PBS and fixed in methanol-Carnoy’s solution overnight. Paraffin embedded sections were stained with DAPI and visualized with a LSM 510 Meta confocal microscope (Carl Zeiss).

### Mucus thickness measurement

*In vivo* mucosal thickness was measured as described previously^38^, with slight modification. The distal colon was cut open longitudinally and the surface of the mucus layer was visualized by adding a suspension of charcoal powder in PBS. A siliconized glass micropipette (1 μm tip diameter) was held by a micromanipulator (KITE-R, World Precision Ins) connected to a digimatic micrometer head (350–251, Mitutoyo), and the entire mucus thickness (Total) was measured under a dissecting microscope (SMZ1000, Nikon). After aspiration of the soluble mucus, the remaining mucus (Firm) layer was measured. The average thickness was calculated by measuring at least ten different spots per mouse in a blind fashion.

### Fluorescence *in situ* hybridization (FISH) for bacteria localization

FISH in the distal colonic section was performed as described previously^38^, except for using 10 μg of an Alexa Fluor 488-conjugated bacterial rRNA EUB338 probe for hybridization. The epithelial region in contact with the bacteria was evaluated on a FISH-stained section, and the proportion of epithelial region in contact with bacteria was determined from a whole intestinal section in a blind fashion using NIS-Elements BR 3.2 imaging software (Nikon). For co-immunostaining, the FISH-stained slides were blocked and incubated with anti-Muc2 antibody (sc-15334, Santa Cruz Biotech.) overnight at 4 °C. The slides were further incubated with goat anti-rabbit Alexa 568 (Life Technologies), counterstained with DAPI solution (0.5 μg/ml in PBS) and visualized with a confocal microscope.

### Statistical analysis

The values are expressed as the mean ± SEM. The quantification data were assessed using a two-tailed unpaired *t*-test with GraphPad Prism program and each *p*-value is marked in the figure. A *p*-value under 0.05 is considered to be statistically significant.

## Additional Information

**How to cite this article**: Lee, S.-y. *et al*. Arhgap17, a RhoGTPase activating protein, regulates mucosal and epithelial barrier function in the mouse colon. *Sci. Rep*. **6**, 26923; doi: 10.1038/srep26923 (2016).

## Supplementary Material

Supplementary Information
