# Supplementary material for: Arhgap17, a RhoGTPase activating protein, regulates mucosal and epithelial barrier function in the mouse colon
Source: Sci Rep. 2016 May 27;6:26923. doi: 10.1038/srep26923 (PMC4882514; doi:10.1038/srep26923)
Supplement: Supplementary Information [file srep26923-s1.pdf]

Arhgap17, a RhoGTPase activating protein, regulates mucosal and epithelial barrier function in the mouse colon

So-young Lee,[1] Hwain Kim,[1] Kyoungmi Kim,[1] Hyunji Lee,[1] Seungbok Lee,[2] and Daekee Lee[1,*]

Supplementary Methods

Identification of Arhgap17$^{Gt(RRN062)Byg}$ allele and generation of Arhgap17 mutant mice

Gene trap ES cell line, Gt(RRN062)Byg (129P2/OlaHsd origin), was obtained from International Gene Trap Consortium. Using genomic DNA of ES cells as a template, the exact structure of *Arhgap17*$^{Gt(RRN062)Byg}$ allele was identified by PCR sequencing. For 5' junction identification, 5'-GACCGAAGTCCTCAGTGAAGATC-3' (forward) specific for exon 2 of *Arhgap17* and 5'-GGGAAAGAGGGCTCTGTCCT-3' (reverse) for gene trap vector (*pGT0lxf*) were used for PCR primers. For 3' junction identification, 5'-CTCACCGGCTCCAGATTTATCA-3' (forward) for *pGT0lxf* and 5'-CGTCAGAGCTCTAGGCTGGA-3' (reverse) for *Arhgap17* were used for PCR primers. PCR sequencing of *Arhgap17*$^{Gt(RRN062)Byg}$ allele of ES cells revealed that splicing acceptor from gene trap vector was inserted into intron 2 of *Arhgap17* (Supplementary Figure 1A). Sequencing of RT-PCR product of gene trap allele showed that exon 2 of *Arhgap17* was followed by En2 coding leader in gene trap vector as expected, which would code for fusion protein tagging with 31 amino acids of Arhgap17 N-terminal region.

ES cells were injected into C57BL/6J-derived blastocysts to generate chimeric mice and chimeric mice were further bred to C57BL/6J female to obtain heterozygous mice. Heterozygous mice were maintained congenic on C57BL/6J background more than 10 generations. Genotypes were determined by PCR with three primers (Supplementary Figure 1A): Arhgap17-S1, 5'-GAGATGCATGATGGGATTGCG-3' (forward); Arhgap17-AS1, 5'-CGTCAGAGCTCTAGGCTGGA-3' (reverse); En2-AS1, 5'-CCGGAGCGGATCTCAAACTC-3' (reverse). Arhgap17-S1 and Arhgap17-AS1 amplify the wild-type allele and Arhgap17-S1 and En2-AS1

amplify the mutant allele, respectively. Genotypes of offspring were determined by PCR followed by agarose gel electrophoresis (Supplementary Figure 1B). Homozygous mutant mice with *Arhgap17*$^{\mathrm{Gt(RRN062)Byg}}$ allele (here it named *Arhgap17*^{m}) were produced at Mendelian ratios from heterozygous intercrosses and developed normally. RT-PCR analysis using three primers (Supplementary Figure 1A) to detect endogenous *Arhgap17* and mutant mRNA expression showed that endogenous *Arhgap17* mRNA was not expressed in tissues of homozygous mice (Supplementary Figure 1C). Whether any *Arhgap17* expression occurred by alternative splicing from *Arhgap17*^{m} allele, qRT-PCR was performed using far downstream primers for exon 11 (forward) and exon 13 (reverse). The mRNA levels were approximately one-half in the heterozygous mice and less than 1% in homozygous mutant mice compared to wild-type mice (Supplementary Figure 1D), respectively. Moreover, immunoprecipitation-western blotting exhibited no protein band around 95 kD from the homozygous mutant mice (Supplementary Figure 1E), suggesting that *Arhgap17*^{m} allele is likely a null allele. Then, homozygous mutant (*Arhgap17*$^{m/m}$) mice are stated as arhgap17-deficient mice.

RNA purification and RT-PCR

Total RNA was isolated from tissues using Trizol reagent (Life Technologies) as suggested by manufacturer. Reverse transcription and PCR were performed as described previously.[1] The primers for PCR are Arhgap17-E2, 5’-GACCGAAGTCCTCAGTGAAGATC-3’ (forward); Arhgap17-E4, 5’-TGTTCTGGGCAAGAGCTGTCA-3’ (reverse); β-geo, 5’-GTATCGGCCTCAGGAAGATCG-3’ (reverse). Arhgap17-E2 and Arhgap17-E4 amplify cDNA of the endogenous mRNA, and Arhgap17-E2 and β-geo amplify cDNA of the mutant mRNA, respectively.

The primer sequences for qRT-PCR

18S rRNA: 5’-TCAACTTTCGATGGTAGTCGCC-3’ and 5’-GGCCTCGAAAGAGTCCTGTATTGT-3’

Arhgap17: 5’-AGCTGAAAGCTGCTCTCGACTGC-3’ and 5’-TGGCGGCAACTTCTGACATGTT-3’

Cxcl1: 5’-AATGAGCTGCGCTGTCAGTG-3’ and 5’-TGGGGACACCTTTTAGCATC-3’

Cxcl2: 5’-CCAGTGAACTGCGCTGTCAA-3’ and 5’-GTTAGCCTTGCCTTTGTTCAG-3’

Gapdh: 5’-GGAAGGGCTCATGACCACA-3’ and 5’-CAGTGAGCTTCCCGTTCAG-3’

Ifng: 5’-AGGAACTGGCAAAAGGATGG-3’ and 5’-TGTGGGTTGTTGACCTCAAA-3’

Il1b: 5’-CGGACCCATATGAGCTGAAA-3’ and 5’-CTTGGGATCCACACTCTCCA-3’

Il6: 5’-ATGAGAAAAGAGTTGTGCAATGGC-3’ and 5’-CCAGGTAGCTATGGTACTCCAGAA-3’

Il10: 5’-TACTGCTAACCGACTCCTTAATG-3’ and 5’-AGGGGAGAAATCGATGACAG-3’

Il12a: 5’-TCAGAAGCTAACCATCTCCTGG-3’ and 5’-CTCCATGTCTCTGGTCTGAGG-3’

Muc2: 5’-ACTCATGGTGGAGCTGGACA-3’ and 5’-CCGGTTCTTGTACTGCCTCA-3’

Tff3: 5’-ATTACGTTGGCCTGTCTCCA-3’ and 5’-GCCACGGTTGTTACACTGCTC-3’

Tnf: 5’-GTCCCCAAAGGGATGAGAAGTTC-3’ and 5’-ATCGGCTGGCACCACTAGTTGG-3’

Cell proliferation and apoptosis in the intestine

Tissues were labeled with 10 mM BrdU (Sigma-Aldrich) for 2 hours and flushed with PBS before collection of tissues. The paraffin sections were immunostained with a BrdU staining kit or a TUNEL staining kit as described previously.[2] BrdU or TUNEL stained slides were examined under ECLIPSE 80i light microscope (Nikon) and analyzed with NIS-Elements BR 3.2 imaging software. Twenty crypts per mouse were assessed for analysis.

Histological scoring after DSS treatment

The severity of mucosal injury was graded on a scale of 0 to 3: grade 0, normal; grade 1, partial destruction of crypts; grade 2, complete loss of crypts; and grade 3, complete loss of crypts and epithelial cells.[1,2] The histological score was determined by multiplying the portion of injured surface by the grade of severity. Two different regions of proximal, middle and distal colon were used for histological scoring, and the histological scores from individual mice were determined by adding all six values. Histological scoring was performed in a blinded fashion.

Immunoprecipitation (IP) and western blot analysis

The extract of intestinal tissues was prepared by homogenization in RIPA buffer (50 mM Tris-HCl, pH 8, 150 mM NaCl, 1% lgepal CA-630, 0.5% sodium deoxycholate and 0.1% SDS) supplemented with protease inhibitors (1 mM phenylmethylsulfonyl fluoride, 10 μg/ml leupeptin and 10 μg/ml aprotinin) and phosphatase inhibitors (1 mM Na_3VO_4, 1 mM NaF and 10 mM β-glycerophosphate). The cleared extract (300 μg) was adjusted to 0.8 ml with HNTG buffer (20 mM HEPES, pH 7.4, 150 mM NaCl, 0.1% Triton X-100 and 10% glycerol) containing protease and phosphatase inhibitors. The mixture was incubated with 2 μg of arhgap17 (sc-160146, Santa Cruz Biotech) antibody overnight at 4°C, followed by incubation with 20 μl of protein G beads (Pierce) for 3 hours at 4°C. The mixture was washed with HNTG buffer 4 times, eluted with 20 μl of 2× SDS sample buffer by heating 95°C for 3 min and used for western blotting as described previously,[1] using anti-arhgap17 antibody (sc-160145, Santa Cruz Biotech). For straight western blotting, tissue collection, homogenization, protein quantification, SDS-PAGE, western blotting and quantification of blots were performed as described previously.[2] The antibodies used were: β-actin (AC-15, Sigma-Aldrich); anti-Tff3 antibody (kindly from Dr. Nam).

LacZ staining

The *Arhgap17* expression in tissues was determined by β-galactosidase (LacZ) staining. For whole mount LacZ staining, embryos were fixed in 0.25% glutaraldehyde, 5 mM EGTA and 2 mM $MgCl_2$ in PBS for 10 minutes at 4°C. After rinsing with detergent solution (2 mM $MgCl_2$, 0.02% Igepal CA-630, 0.01% sodium deoxycholate in PBS) three times, embryos were stained with X-gal staining solution[1] for 2 to 3 hours at 37°C and examined under dissecting microscope. To analyze the endogenous *Arhgap17* expression in adult, tissues collected from arghap17-deficient mice were fixed, frozen-sectioned and stained with X-gal as described previously.[1] The X-gal-stained tissues were counterstained with 0.1× (v/v) Harris hematoxylin (Sigma-Aldrich) followed by eosinY staining not to disturb the X-gal staining. The image was visualized with light microscope.

The primary antibodies for immunofluorescence and immunohistochemistry

The primary antibodies were anti-E-cadherin (610181, BD Biosciences), anti-*β*-catenin antibody (sc-7199, Santa Cruz Biotech), anti-occludin antibody (711500, Life Technologies), anti-ZO-1 (402200, Life Technologies), anti-TNF antibody (ab6671, Abcam), anti-Tff3 antibody (kindly from Dr. Nam). F-actin was stained with Alexa 568-conjugated Phalloidin (A12380, Life Technologies).

Co-immunofluorescence staining of E-cadherin and F-actin

Frozen sections were blocked in M.O.M mouse Ig blocking reagent (Vector M.O.M Immunodetection kit, Vector lab) for 1 hour at room temperature and treated with anti-E-cadherin antibody in M.O.M diluent solution at 4°C overnight. The sections were washed 4 times with TBST and

treated with secondary antibodies conjugated with Alexa 488 and Phalloidin Alexa 568 (A12380, Life Technologies) for 1 hour at room temperature. Sections were counterstained with DAPI and visualized with a confocal microscope. The data was performed by Z-section per 1 μm on sample.

Migration of epithelial cells in the intestine

Tissues were labeled with 10 mM BrdU for 30 hours and flushed with PBS before collection of tissues. The paraffin sections were immunostained with a BrdU staining kit and cell migration was analyzed by measuring the length of far upstream BrdU-stained epithelial cells from the crypt using NIS-Elements BR 3.2 imaging software (Nikon). Twenty villus or ten crypts per mouse were assessed for analysis.

Supplementary Figure Legends

Supplementary Figure 1. Structure of the *Arhgap17* gene trap locus and analysis of Arhgap17 expression. (A) Exon 2 to exon 4 of *Arhgap17* are labeled 2 to 4. The gene trap vector, pGT0lxf, consists of an *En2* splicing acceptor sequence (SA), β-geo (*lacZ* fused with neomycin transphosphorylase fusion gene) and SV40 poly(A) site (pA). The arrows indicate primers for PCR genotyping, and the arrowheads indicate primers for RT-PCR. (B) Genotypes of offspring were determined by PCR and agarose gel electrophoresis (a 271-bp product for wild-type; a 171-bp product for mutant allele; M, 1 kb plus DNA ladder from Life Technologies). (C) RT-PCR analysis of the endogenous *Arhgap17* and mutant mRNA expression from the indicated tissues of wild-type, heterozygote and mutant mice (a 170-bp product for the wild-type; a 359-bp product for the mutant allele; M, 1 kb plus DNA ladder). (D) qRT-PCR of *Arhgap17* mRNA expression in the distal colon of wild-type, heterozygote and mutant mice. (E) IP-western blot for Arhgap17 protein in the small intestine from wild-type and mutant mice. NS indicates non-specific bands.

Supplementary Figure 2. Examination of Arhgap17 expression by LacZ staining in various tissues. (A) Whole mount LacZ staining was performed on embryonic day 10.5 of wild-type (right) and *Arhgap17*$^{m/m}$ (left) embryos. (B) Frozen sections were LacZ stained in various tissues. a, hippocampus; b, heart; c, lung; d, esophagus; e, stomach; f, muscle; g, kidney; h, testis; i, skin. Scale bar, 50 μm.

Supplementary Figure 3. Analysis of intestinal epithelial cell proliferation, apoptosis, and

migration in arhgap17-deficient mice. (A) The ileum sections were immunostained with an anti-BrdU antibody or analyzed with the TUNEL assay. (B) The number of BrdU-positive cells or TUNEL-positive cells (arrows) per crypt are shown (BrdU, n = 5; TUNEL, n = 5). +/+, wild-type; *m/m*, arhgap17-deficient mice. (C) BrdU-injected mice were sacrificed 30 hours after BrdU treatment, and intestinal sections were immunostained with an anti-BrdU antibody to analyze cell migration. (D) Cell migration was measured in wild-type and arhgap17-deficient mice, n = 5. Scale bar, 50 μm.

Supplementary Figure 4. Changes in body weight. Body weight was measured weekly in wild-type (+/+) and arhgap17-deficient mice (*m/m*). (A) Male; n = 6. (B) Female; n = 5. Two-way ANOVA for the effect of genotype on body weight was performed.

Supplementary Figure 5. Examination of Arhgap17 expression and analysis of cell proliferation, apoptosis, and migration after DSS treatment for 2 days. (A) Arhgap17 expression was examined by LacZ staining after DSS treatment in arhgap17-deficient mice. The distal colon was collected at the indicated days after DSS treatment, and frozen sections were stained with LacZ staining. Top, original magnification, 100×; Bottom, 200× of dashed rectangles. (B) The distal colon were collected 2 days after DSS treatment, and analyzed with an anti-BrdU antibody or TUNEL assay. For cell migration assay, BrdU-injected mice were sacrificed 30 hours after BrdU treatment, and sections were immunostained with an anti-BrdU antibody. (C) The number of BrdU-positive cells or TUNEL-positive cells (arrows) per crypt and the relative cell migration are shown (BrdU, n = 6; TUNEL, n = 9 to 11; Migration, n = 6). +/+, wild-type; *m/m*, arhgap17-deficient mice. Scale bar, 50 μm.

Supplementary Figure 6. Immunofluorescence analysis of TJs and AJs proteins in the ileum (A, B) before or (C, D) after DSS treatment for two days. (A, C) Sections were immunostained with TJ markers, an anti-occludin antibody and an anti-ZO-1 antibody. The arrows indicate the mislocalization of TJ proteins in the apical/lateral border of epithelial cells, n = 2 to 3. (B, D) Sections were immunostained with AJs markers, an anti-E-cadherin antibody and an anti-*β*-catenin antibody, n = 3 to 7. The arrows indicate the loss of junction proteins in the apical/lateral boarder of epithelial cells, whereas the arrowheads indicate lumps of junction proteins in the basolateral epithelium. The villus is shown on the left, and the crypt is shown on the right. Scale bar, 20 μm.

Supplementary Figure 7. Immunofluorescence staining of E-cadherin in the apical/lateral border of arhgap17-deficient mice. (A) The distal colon and (B) ileum were analyzed by co-immunofluorescence staining with an anti-E-cadherin antibody to reveal the basolateral membrane and with Phalloidin to stain the apical surface of epithelial cells. Left, original magnification, 400×. Scale bar, 50 μm; Middle and Right, 1200× magnification of a rectangle. Scale bar, 10 μm. Far right panels represent a cross section of the part shown in white dashed rectangle. The arrows indicate weak E-cadherin staining in the apical/lateral boarder. A, n = 5; B, n = 3.

Supplementary Figure 8. (A) The mice were treated for two days with DSS and the mucosal barrier in the distal colon was analyzed by hybridization with the EUB338 probe (green), followed by DAPI staining (blue). Asterisks indicate bacteria that are in contact with the epithelial cells. Original magnification, 100×; Top left, 400× magnification of a dashed rectangle in arhgap17-deficient mice. Scale bar, 50 μm, n = 8. (B) Total RNA was extracted from the distal colon of indicated mice before

DSS treatment and *Tff3* mRNA levels were determined using qRT-PCR, n = 12.

Supplementary References

1 Lee, D. *et al.* Epiregulin is not essential for development of intestinal tumors but is required for protection from intestinal damage. *Molecular and cellular biology* **24**, 8907-8916 (2004).

2 Lee, D. *et al.* Tumor-specific apoptosis caused by deletion of the ERBB3 pseudo-kinase in mouse intestinal epithelium. *The Journal of clinical investigation* **119**, 2702-2713 (2009).

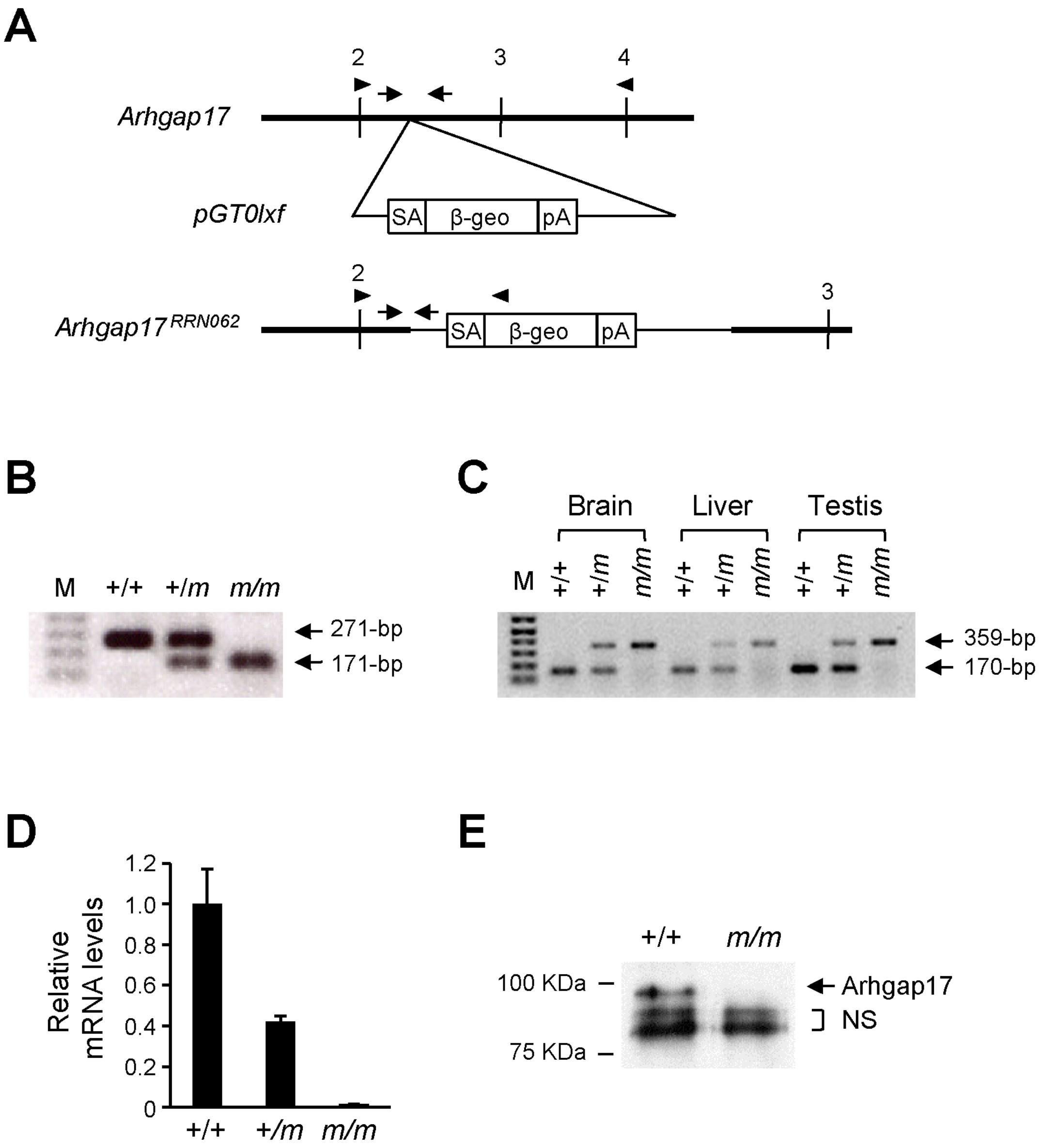

Supplementary Figure 1

A

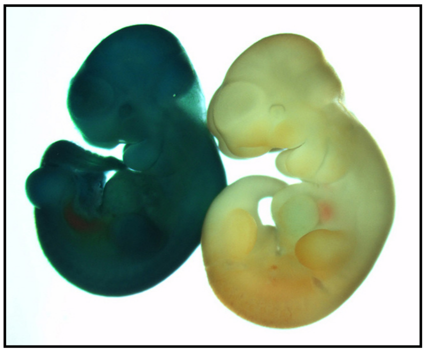

B

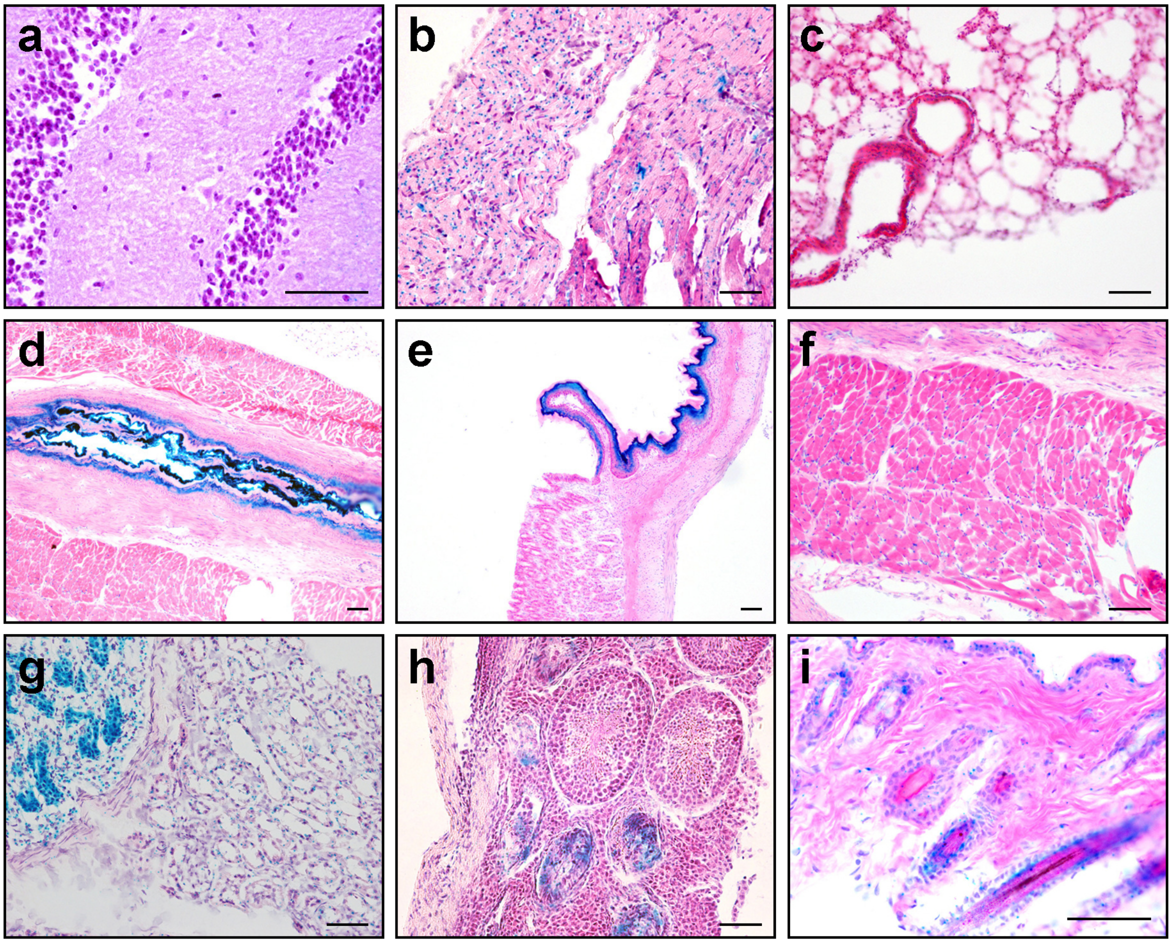

Supplementary Figure 2

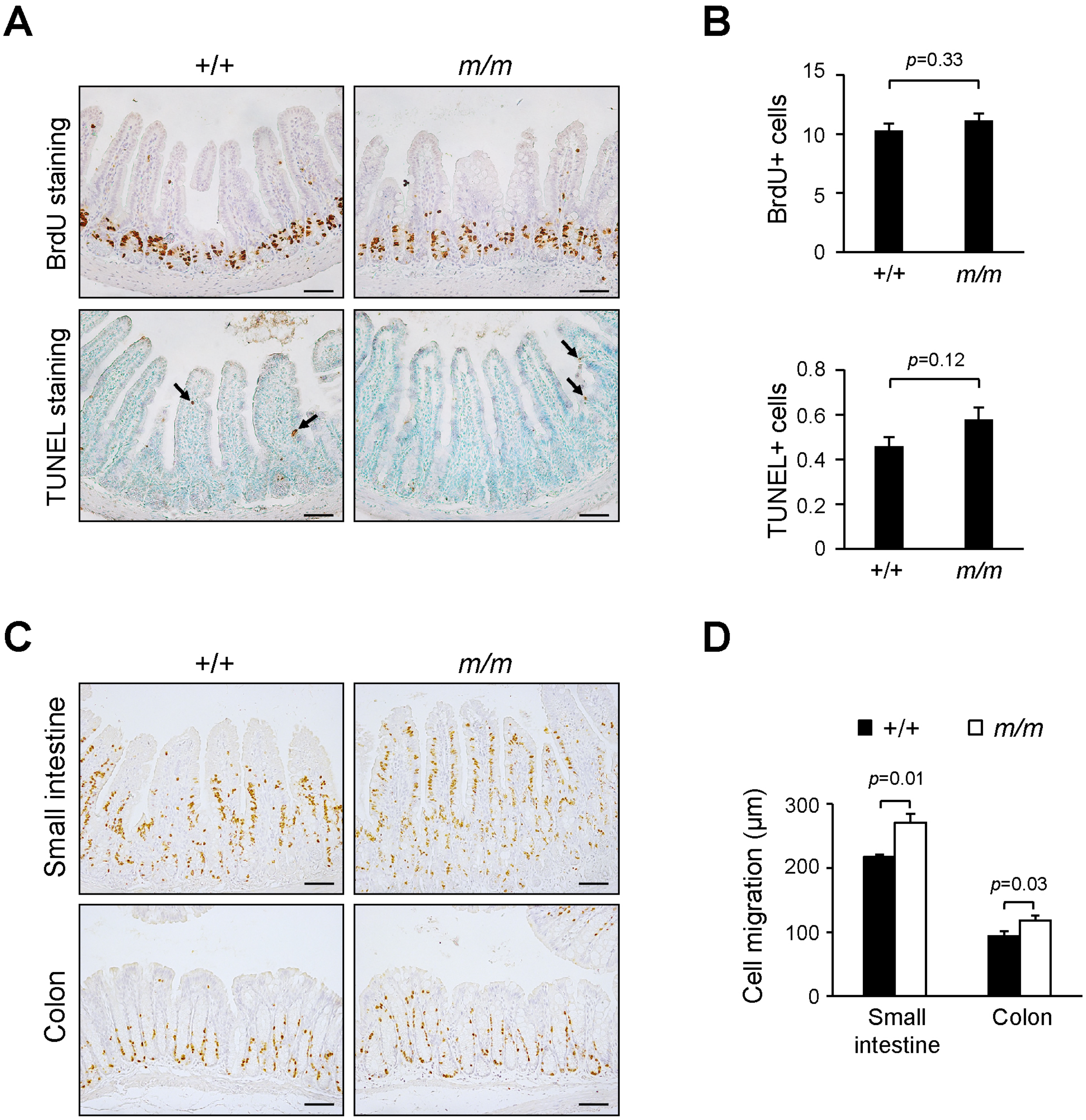

Supplementary Figure 3

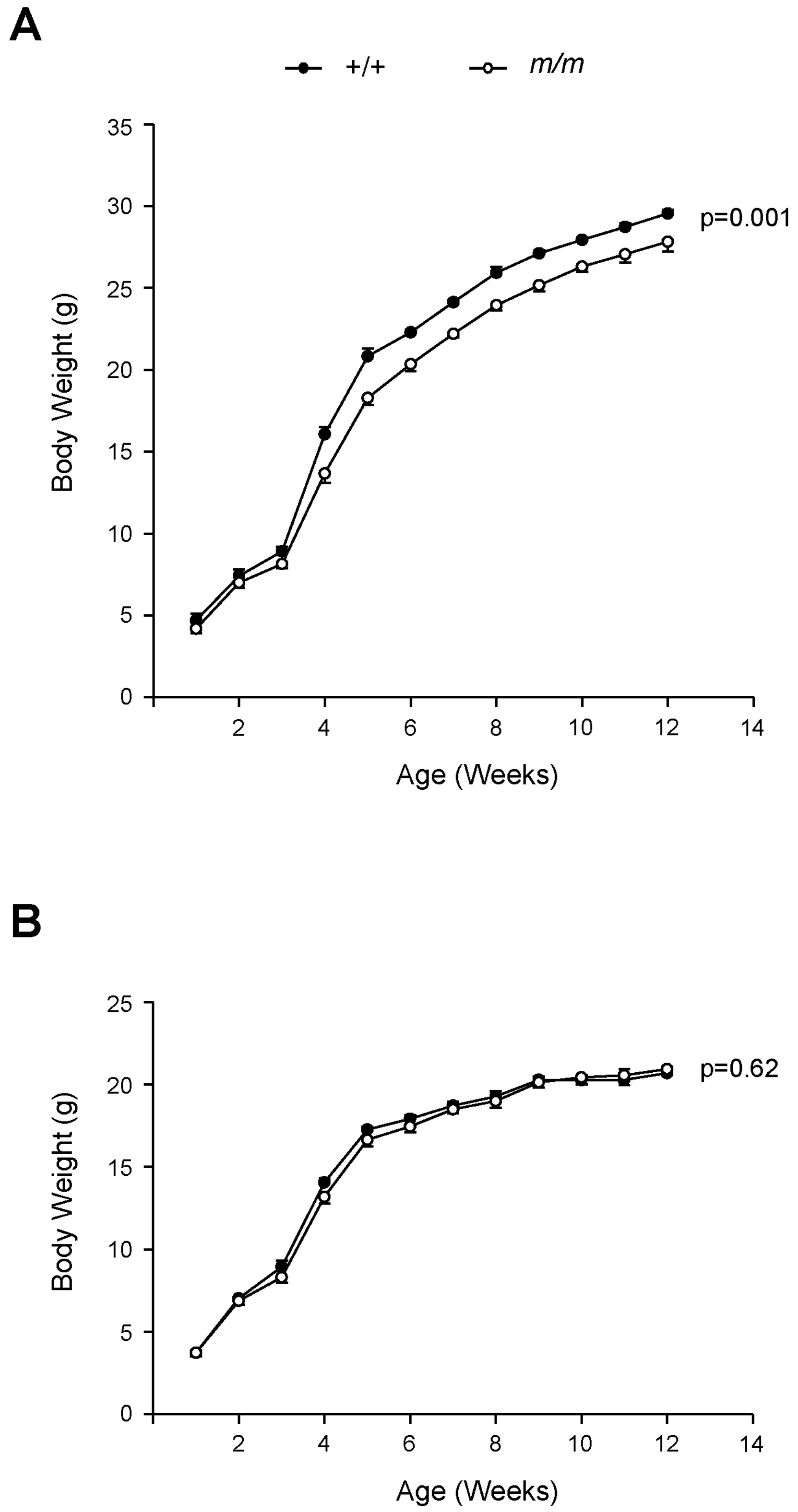

Supplementary Figure 4

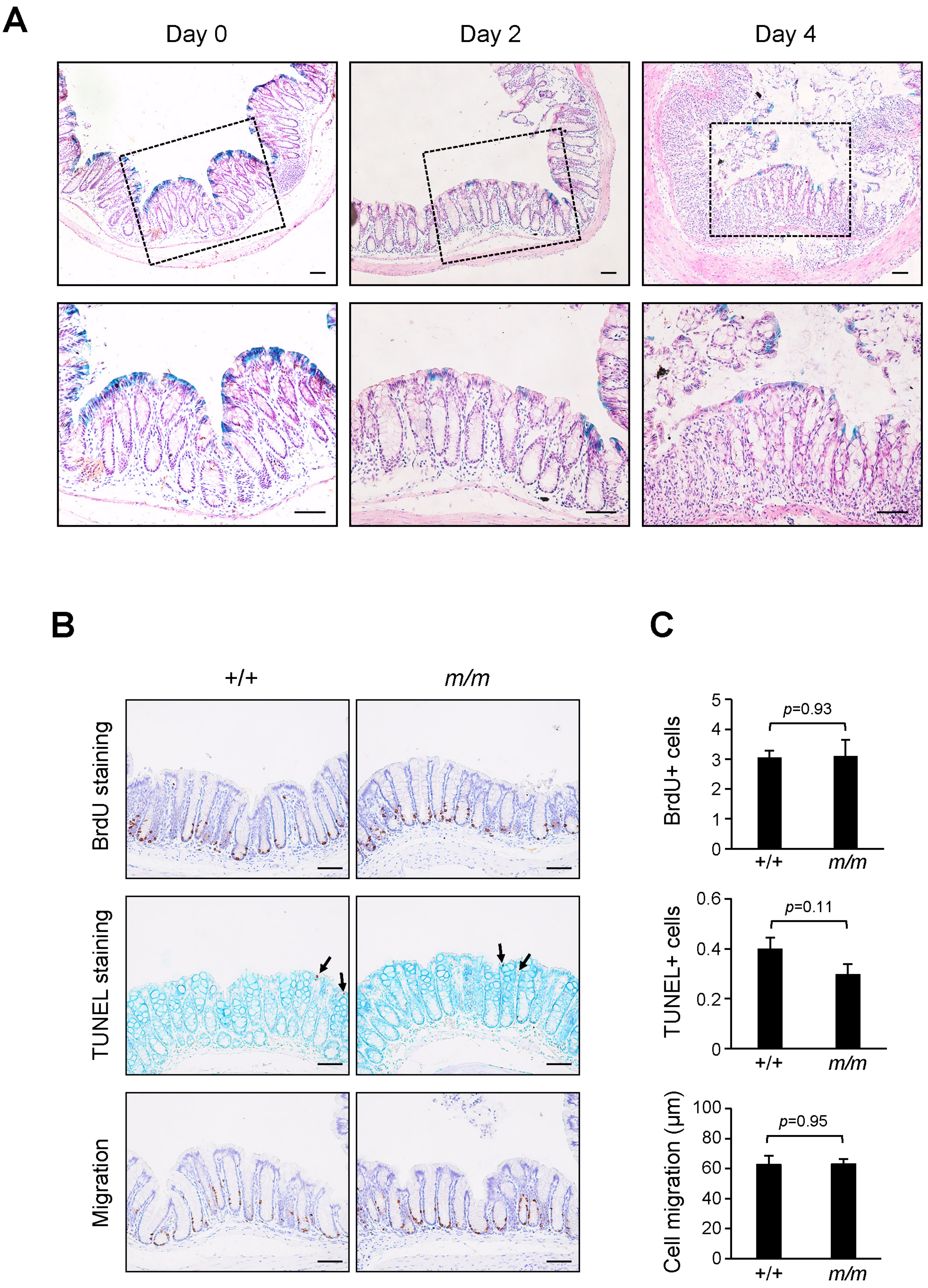

Supplementary Figure 5

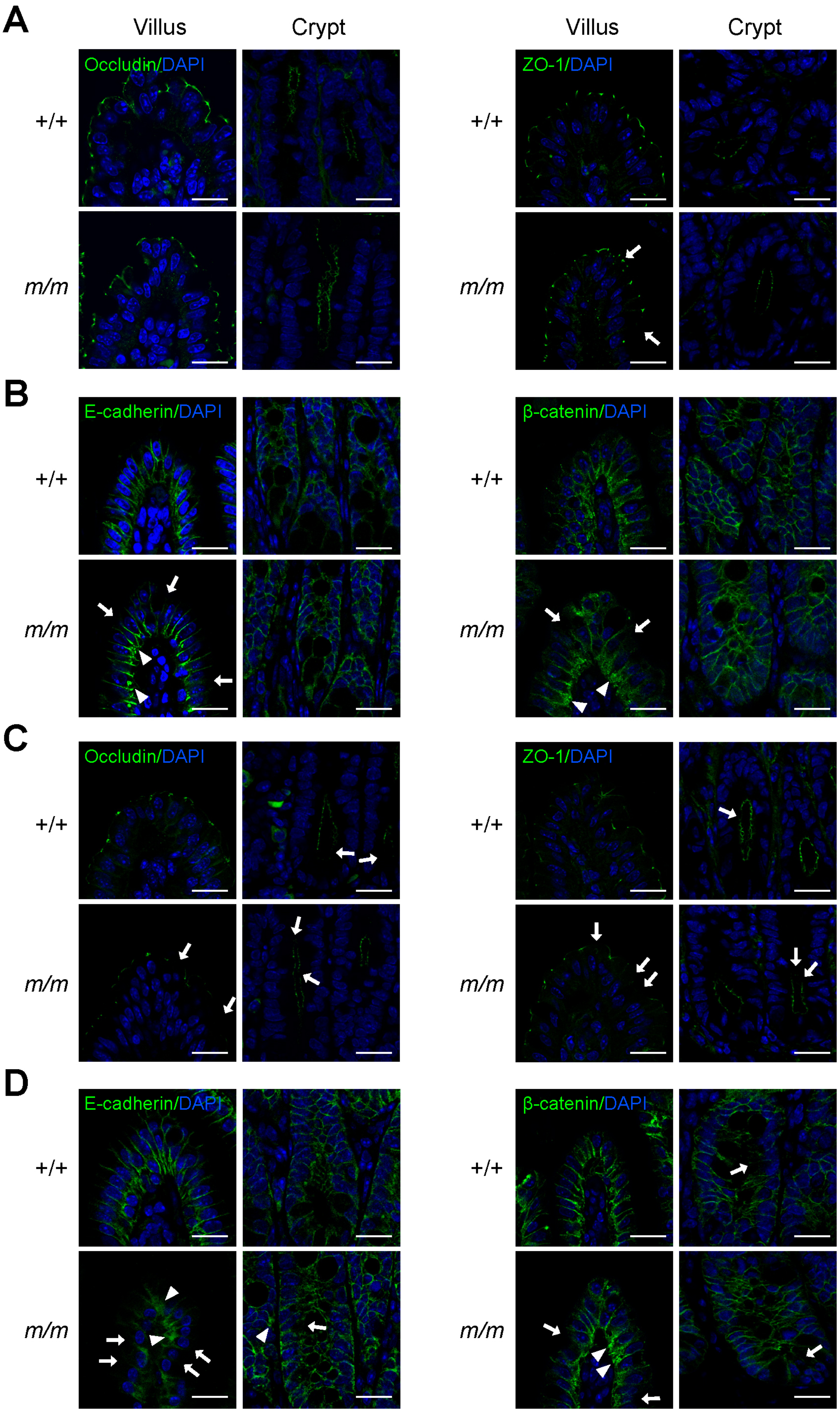

Supplementary Figure 6

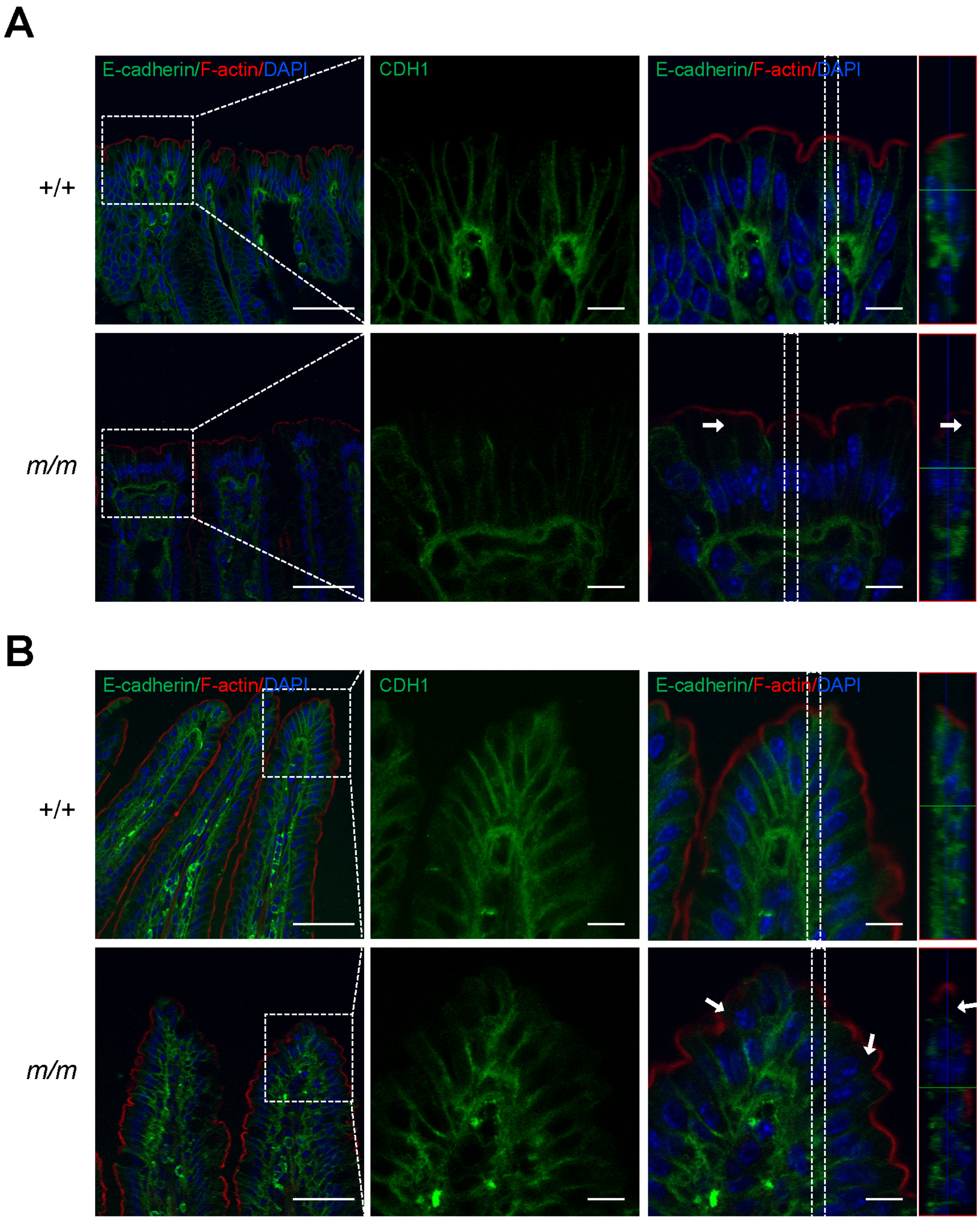

Supplementary Figure 7

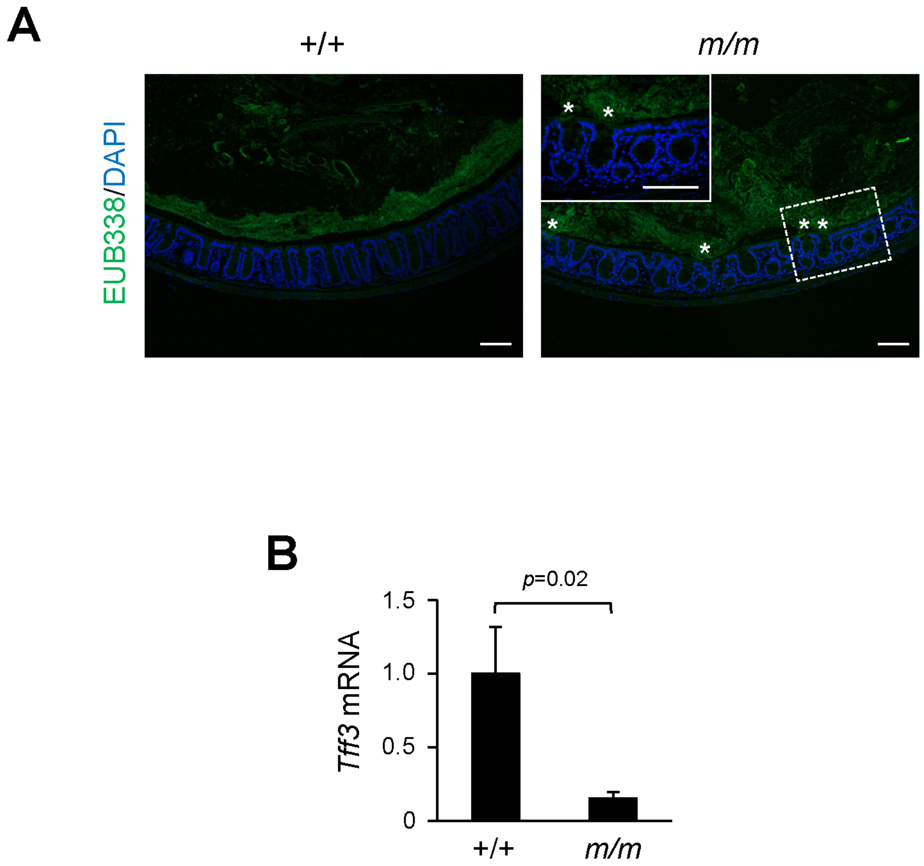

Supplementary Figure 8
